# Comparative Study on “Long-Dan”, “Qin-Jiao” and Their Adulterants by HPLC Analysis

**DOI:** 10.1007/s13659-014-0039-x

**Published:** 2014-10-04

**Authors:** Fang-Fang Liu, Yan-Ming Wang, Hong-Tao Zhu, Dong Wang, Chong-Ren Yang, Min Xu, Ying-Jun Zhang

**Affiliations:** 1 State Key Laboratory of Phytochemistry and Plant Resources in West China, Kunming Institute of Botany, Chinese Academy of Sciences, Kunming, 650204 People’s Republic of China; 2 Yunnan University of Traditional Chinese Medicine, Kunming, 650500 People’s Republic of China; 3 University of Chinese Academy of Sciences, Beijing, 100049 People’s Republic of China

**Keywords:** “Long-Dan”, “Qin-Jiao”, *Gentiana*, HPLC analysis, Iridoid glycosides

## Abstract

“Long-Dan” and “Qin-Jiao” are two important TCM herbs since ancient times in China. In the Chinese Pharmacopoeia, the dried roots and rhizomes of four species from the genus *Gentiana*, e.g. *Gentiana manshurica*, *G. scabra*, *G. triflora* and *G. rigescens*, are recorded under the name of Gentianae Radix et Rhizoma (“Long-Dan” in Chinese), while the other four species from the same genus including *G. macrophylla*, *G. crassicaulis*, *G. straminea* and *G. duhurica* are recorded and used as the raw materials of Gentianae Macrophyllae Radix (“Qin-Jiao” in Chinese). On the basis of the establishment of a validated HPLC–UV method for quantifying simultaneously, five iridoid glycosides, e.g. loganic acid (**1**), swertiamarinin (**2**), gentiopicroside (**3**), sweroside (**4**) and 2′-(*o*,*m*-dihydroxybenzyl)sweroside (**5**) have been used successfully as chemical markers for the comparison of the species used as “Long-Dan”, “Qin-Jiao” and their adulterants in the present study. The results suggested that four iridoid glycosides **1**–**4** commonly existed in both “Long-Dan” and “Qin-Jiao”, while 2′-(*o*,*m*-dihydroxybenzyl)sweroside (**5**) also existed as one of the major components in “Dian-Long-Dan” species. Moreover, the contents of compounds **1**–**5** were various in different “Long-Dan” and “Qin-Jiao” species. Herein, we profiled and compared three “Long-Dan” species, four “Qin-Jiao” species and five adulterants by applying multivariate statistical techniques to their HPLC data sets to establish the differences and/or similarities.

**Electronic supplementary material:**

The online version of this article (doi:10.1007/s13659-014-0039-x) contains supplementary material, which is available to authorized users.

## Introduction

In China, “Long-Dan” is typically used for protecting liver [1], and is commonly used for curing inflammation, hepatitis, rheumatism, cholecystitis and tuberculosis as a well-known traditional Chinese medicinal (TCM) herb [2]. While, “Qin-Jiao”, another important TCM herb for fighting rheumatism since ancient times in China, has been used as therapy for rheumatism, arthralgia, stroke, hemiplegia, pains, jaundice and infantile malnutrition [3, 4]. The original plants of both “Long-Dan” and “Qin-Jiao” are from the genus *Gentiana* (Gentianaceae). From which, the dried roots and rhizomes of four species, e.g. *Gentiana manshurica*, *G. scabra*, *G. triflora* and *G. rigescens*, are recorded under the name of Gentianae Radix et Rhizoma (“Long-Dan” in Chinese) in the Chinese Pharmacopoeia, while the other four species including *G. macrophylla*, *G. crassicaulis*, *G. straminea* and *G. duhurica* are used as the raw materials of Gentianae Macrophyllae Radix (“Qin-Jiao” in Chinese). In addition to these eight species, most of the *Gentiana* plants, e.g. *G. purdomii*, *G. microdonta*, *G. obconica*, *G. erecto*-*sepala*, *G. robusta,* have been used as ethno-medicines for “Long-Dan” or “Qin-Jiao” by the local people living in their distributing areas. [5–8].

In general, the qualities and chemical compositions of herbs vary widely, depending substantially on their different species, variety, geographical origin, cultivation, environment, and so on. It was considered that the qualities and chemical compositions of “Long-Dan” and “Qin-Jiao” could be significantly affected by such factors. Different “Long-Dan” and “Qin-Jiao” species previously have been chemically and biologically investigated on by several groups [9–12]. The comparative study on “Long-Dan” and related adulterants by HPLC analysis was also developed by Jiang, et al. [13]. Previous studies suggested that loganic acid, gentiopicroside, sweroside and swertiamarinin, existing widely in genus *Gentiana*, were the main compounds in “Long-Dan” and “Qin-Jiao”. Among them, loganic acid could inhibit the carrageenan-induced mouse paw edema [14], and gentiopicroside showed inhibitory effects on inflammatory mediators NO and COX-2 [15]. Our recent study showed that iridoid glycosides as the major constituents in “Qin-Jiao” (*G.dahurica*, *G. crassicaulis* and *G. straminea*) and “Long-Dan” (*G. rigescens*), displayed potential COXs-2/1 inhibitory activities in zebrafish model [12]. However, a detailed comparison among different species used as “Qin-Jiao” and “Long-Dan”, and their related adulterants by applying multivariate statistical techniques is lacking. Herein, a quantitative analysis of five main constituents in *Gentiana* species, e.g., loganic acid (**1**), swertiamarinin (**2**), gentiopicroside (**3**), sweroside (**4**) and 2′-(*o,m*-dihydroxybenzyl)sweroside (**5**) was established, and their profiling and comparison in 39 *Gentiana* samples referring to three “Long-Dan” species, four “Qin-Jiao” species and five other relating adulterants were studied by applying multivariate statistical techniques to their HPLC data sets, in order to establish the differences and/or similarities.

## Results and Discussion

### Identification of *Compounds***1**–**5**

Compounds **1**–**5** were identified by HPLC–DAD–MS analysis, on the basis of their retention time, UV absorption, the quasi-molecular ions, fragment ions, and co-HPLC comparison with authentic standards, as well as the data published previously. In the LC–MS spectra, the retention times and quasi-molecular ions of the five compounds were as follows: *t*_R_ = 5.19 min, *m*/*z* = 375 ([M − H]^−^) for compound **1**; *t*_R_ = 8.03 min, *m*/*z* = 397 ([M + Na]^+^) for compound **2**; *t*_R_ = 10.10 min, *m*/*z* = 379 ([M + Na]^+^) for compound **3**; *t*_R_ = 10.70 min, *m*/*z* = 381 ([M + Na]^+^) for compound **4**; *t*_R_ = 19.85 min, *m*/*z* = 493 ([M − H]^−^) for compound **5**.

### Contents of Marker Compounds in *Gentiana* Samples

**Fig. 1:**
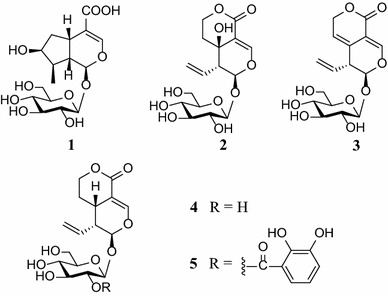
Chemical structures of compounds **1**–**5**

**Fig. 2:**
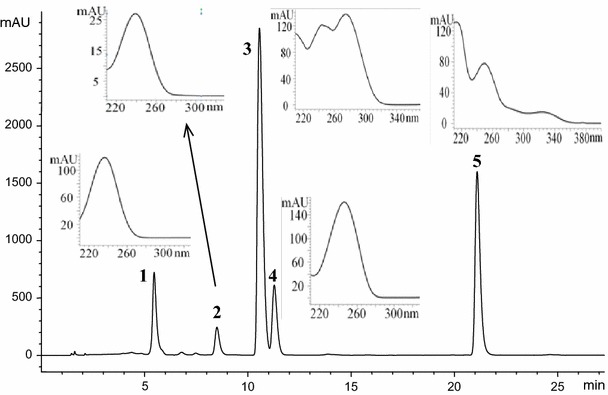
HPLC chromatogram of chemical markers **1**–**5** at 254 nm, and their online UV spectra

The crude methanol extracts of the powdered roots of 39 samples have been prepared, referring to 19 “Long-Dan” samples (S1–S19), seven adulterant samples of “Long-Dan” (S20–S26), 11 “Qin-Jiao” samples (S27–S38) and one adulterant sample of “Qin-Jiao” (S39). The aforementioned samples including 12 different species from 17 different origins were analyzed by HPLC–UV. Table 1 of ESM (SI1) listed the concentration of iridoid glycosides identified in “Qin-Jiao”, “Long-Dan” and their adulterants according to species with their relative peak areas (RPA). Five iridoid glycosides were identified as loganic acid (**1**), swertiamarinin (**2**), gentiopicroside (**3**), sweroside (**4**) and 2′-(*o,m*-dihydroxybenzyl)sweroside (**5**) (Fig. 1), through the comparisons of retention time (*t*_R_) and UV absorption with the standards under the same HPLC conditions (Fig. 2). Among them, gentiopicroside (**3**), one of the main active constituents, was the maximum amount among all the components in both “Long-Dan” and “Qin-Jiao”. The average level of **3** in “Qin-Jiao” (3.51 %) compared with that of them in “Long-Dan” (2.37 %). S19 (*G. triflora*, collected from Qingyuan, Liaoning) possessed the highest content (4.77 %) of **3** among all the “Long-Dan” samples, while the highest content of **3** (6.30 %) was in S27 (*G. crassicaulis*, collected from Diqing, Yunnan) among all the “Qin-Jiao” samples.

**Fig. 3:**
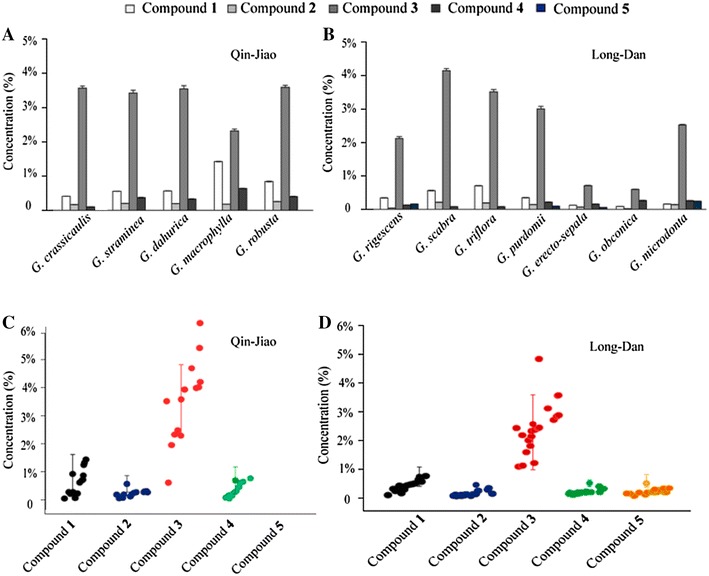
Contents of compounds **1**–**5** in different “Qin-Jiao” and “Long-Dan” species and samples

As for “Long-Dan” samples, the contents of **1**–**4** in *G. scabra* and *G. triflora* were very similar, while those of them in *G*. *rigescens* were similar to their adulterants, *G. purdomii* and *G. microdonta* (Fig. 3B and SI1). The other two adulterants, *G. obconica* (S35) and *G. erecto*-*sepala* (S24) were not qualified medicinally due to their gentiopicroside (**3**) content lower than 2 %, according to the record in the Chinese Pharmacopoeia. It is noted that *G*. *rigescens*, one of the “Long-Dan” species which is also called “Dian-Long-Dan”, is mainly growing in the southwest of China, particularly in the mountainous areas of Yunnan province [16]. Since compound **5** was only detected in *G*. *rigescens*, but not in the other “Long-Dan” species, it could be considered as one of the characteristic components in *G*. *rigescens* [17]. Moreover, among the samples of *G*. *rigescens* collected from different districts of Yunnan, the content of compound **3** in S7 growing in Kunming area possessed the maximum content (3.50 %), while S9 growing in Lijiang had the lowest content (1.04 %).

Among “Qin-Jiao” and its adulterants, the contents of compounds **1**–**4** in *G. crassicaulis*, *G. straminea*, *G. dahurica* and *G. robusta* were quite similar, but higher than those in *G. macrophylla* (Fig. 3A and SI1). Among them, the total contents of compounds **1** and **3** were less than 2.5 % in two samples, S29 and S30 of *G. crassicaulis* (collected from Ganzi in Sichuan provinces, respectively), which could be considered as substandard medicines according to the record in the Chinese Pharmacopoeia. Moreover, the contents of **1** and **3** displayed obviously more different than those of **2** and **4** in different “Qin-Jiao” species (Fig. 3C and SI1).

When comparing of “Long-Dan” with “Qin-Jiao” species, the average contents of compounds **1**–**4** in “Long-Dan” with 0.38, 0.07, 2.37 and 0.13 %, respectively, were lower than those of them in “Qin-Jiao” with 0.61, 0.20, 3.51 and 0.31 %, respectively. Compound **5** was detected only in one “Long-Dan” species, *G*. *rigescens*. The concentrations of compounds **1**–**5** in different “Long-Dan” species displayed more obviously similar than those of them in different “Qin-Jiao” species (Fig. 3D and SI1).

The aforementioned data showed that the qualities and chemical compositions of herbs depend substantially on their different species, varieties, geographical origins, cultivation, environment, and so on. Furthermore, the contents of marker compounds in three adulterants species, *G. purdomii*, *G. microdonta* and *G. robusta*, were quite similar to the samples of “Long-Dan” and “Qin-Jiao”, respectively.

### LC–UV Fingerprint Analysis

**Fig. 4:**
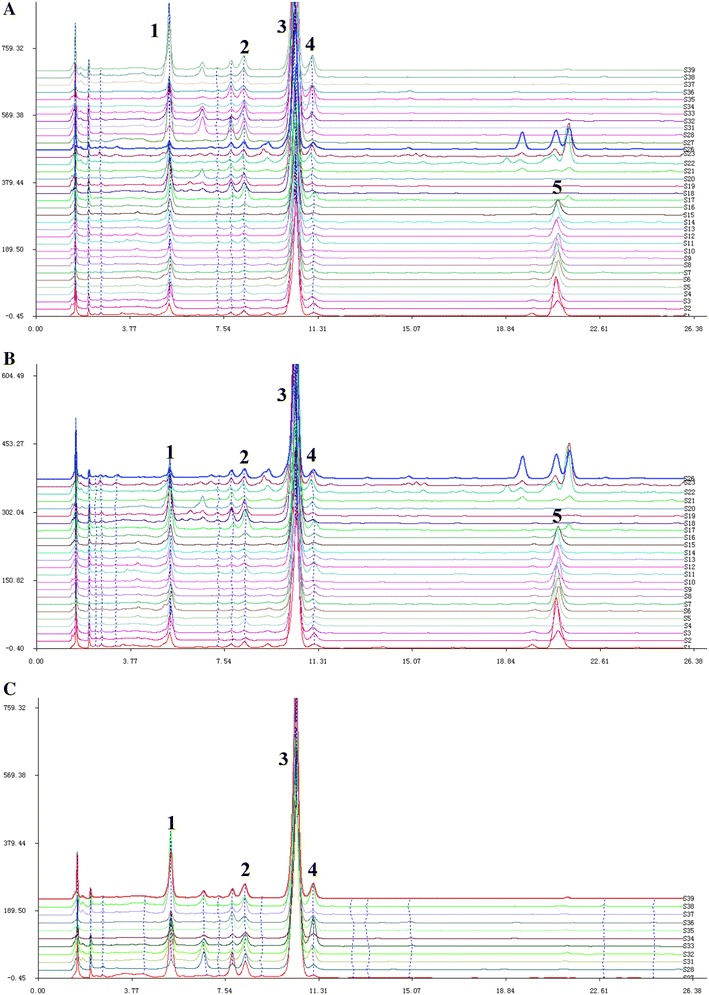
The chromatographic fingerprints of “Long-Dan”, “Qin-Jiao” and their adulterant samples (**A**: the total 35 samples; **B**: “Long-Dan” and its adulterants; **C**: “Qin-Jiao” and its adulterants; **1**: loganic acid; **2**: swertiamarinin; **3**: gentiopicroside; **4**: sweroside; **5**: 2′-(*o,m*-dihydroxybenzyl)sweroside)

**Fig. 5:**
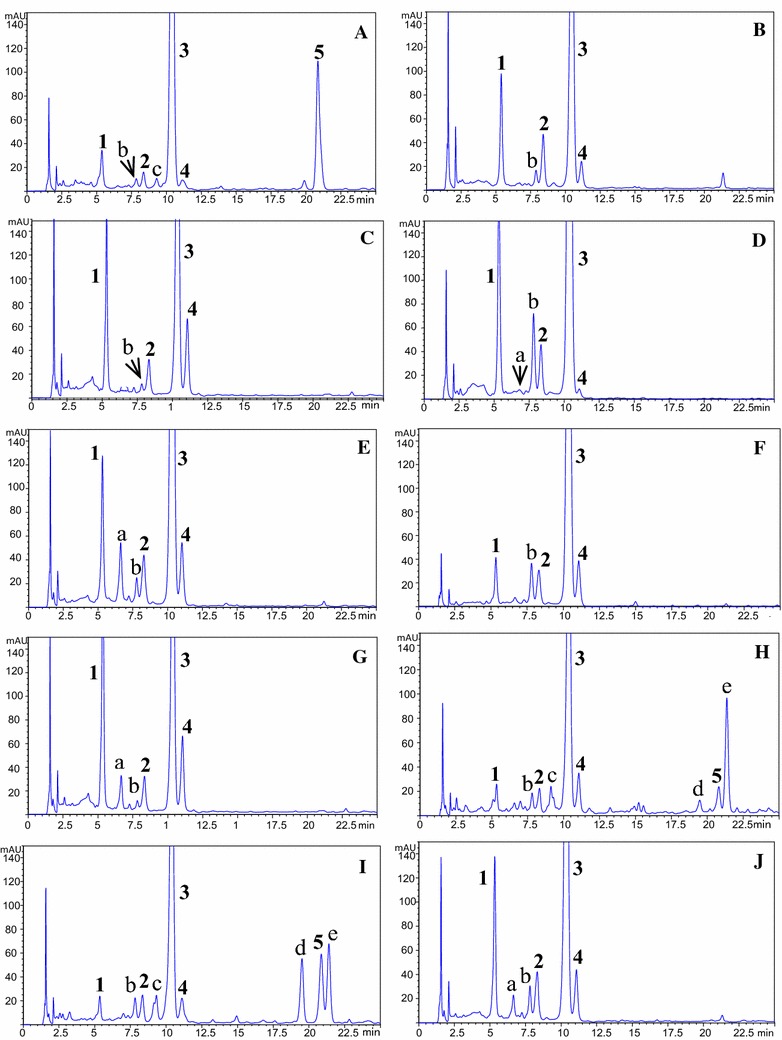
Typical chromatograms of “Long-Dan” (**A**–**C**), “Qin-Jiao” (**D**–**G**) and their adulterants (**H**–**J**) at 254 nm. **A**: *G. rigescens* (S1); **B**: *G. scabra* (S17); **C**: *G. triflora* (S18); **D**: *G. crassicaulis* (S27); **E**: *G. straminea* (S31); **F**: *G. dahurica* (S35); **G**: *G. macrophylla* (S38); **H**: *G. purdomii* (S23); **I**; *G. microdonta* (S26); **J**: *G. robusta* (S39); **1**: loganic acid; **2**; swertiamarinin; **3**: gentiopicroside; **4**: sweroside; **5**: 2′-(*o,m*-dihydroxybenzyl)sweroside

Due to the low content of gentiopicroside (**3**), four samples, *G. crassicaulis* (S24 and S25 from Ganzi in Sichuan provinces, respectively), *G. erecto*-*sepala* (S29), and *G. obconica* (S30) were not included in the following analysis. As shown in Figs. 4A and 5, seven common peaks were showed up in all the 35 samples. Among which, four peaks were identified as loganic acid (**1**), swertiamarinin (**2**), gentiopicroside (**3**) and sweroside (**4**), respectively, by comparing of the t_R_ and UV absorption with those of the standard compounds.

In addition to the seven common peaks, two more peaks (t_R_ = 2.37 and 3.18 min) were observed in 24 samples including “Long-Dan” and its adulterants (*G. purdomii* and *G. microdonta*) (Fig. 4B). The peak at *t*_R_ = 21.0 min identified as 2′-(*o,m*-dihydroxybenzyl)sweroside (**5**) was observed in all the 16 samples of *G. rigescens* (S1–S16). The similarity of all the 24 samples of “Long-Dan” and its adulterants was between 0.939 and 0.996. The HPLC fingerprint chromatograms at 254 nm of “Long-Dan” [*G. rigescens* (S1), *G. scabra* (S17), *G. triflora* (S18)], and its adulterants, *G. purdomii* (S23) and *G. microdonta* (S26) were shown in Fig. 5. As the major components, loganic acid (**1**), swertiamarinin (**2**), gentiopicroside (**3**), and sweroside (**4**) were found in all the species. Three characteristic peaks including 2′-(*o,m*-dihydroxybenzyl)sweroside (**5**) and peaks d–e were all detected in two “Long-Dan” adulterants, *G. purdomii* (S20–S23, Fig. 5H) and *G. microdonta* (S26, Fig. 5I). However, they were not all existed in the other “Long-Dan” samples, suggesting these two adulterants could be distinguished from “Long-Dan” by HPLC analysis.

In the case of 11 “Qin-Jiao” and its adulterants, eight more common peaks (t_R_ = 4.26, 6.67, 8.99, 12.65, 13.27, 14.99, 22.79 and 24.8 min) were observed (Fig. 4C). The similarity indices in 11 samples of “Qin-Jiao” and adulterant samples ranged from 0.960 to 0.999. The HPLC fingerprint chromatograms at 254 nm of “Qin-Jiao” [*G. crassicaulis* (S27), *G. straminea* (S31), *G. dahurica* (S35), *G. macrophylla* (S38)] and its adulterant [*G. robusta* (S39)] were shown in Fig. 5. It is noted that peak a showed in all the “Qin-Jiao” samples and its adulterant *G. robusta*, while not in the “Long-Dan” samples. Although peak a had no identification, the results suggested that peak a was common typical component in “Qin-Jiao” and “Qin-Jiao” could be distinguished from “Long-Dan” by HPLC analysis on peak a except for four major compounds.

Among the tested samples, *G. purdomii* and *G. microdonta* as the adulterants of “Long-Dan” and *G. robusta* as the adulterant of “Qin-Jiao”, contained all the seven common peaks, accounting for more than 90 % of the total peak area. Of them, gentiopicroside (**3**) with all above 60 % of the total peak area displayed the highest content among all the peaks. It suggested that these three species had not only close similarity of chemical compositions, but also similar chromatographic patterns to those of “Long-Dan” and “Qin-Jiao” recorded in the Chinese Pharmacopoeia, respectively. They were considerable to be used respectively as adulterants for “Long-Dan” or “Qin-Jiao”, on the basis of the HPLC–UV fingerprint analysis.

### Hierarchical Clustering Analysis

**Fig. 6:**
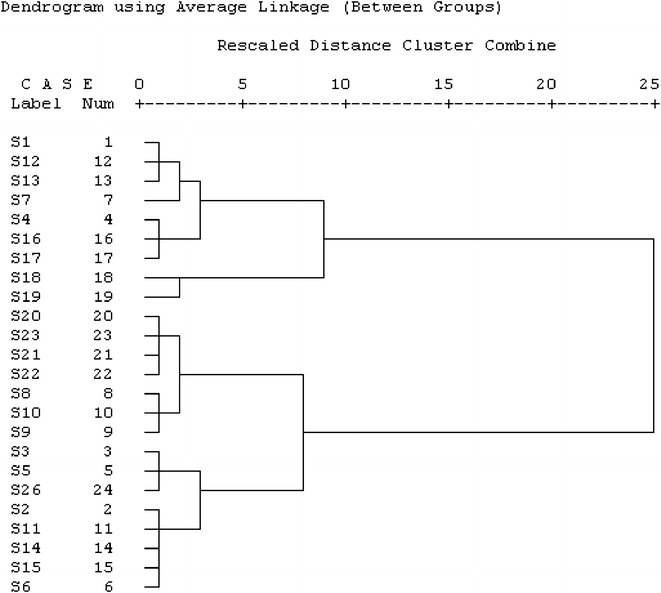
Dendrogram of clustering analysis for “Long-Dan” and its adulterant. (24 samples)

**Fig. 7:**
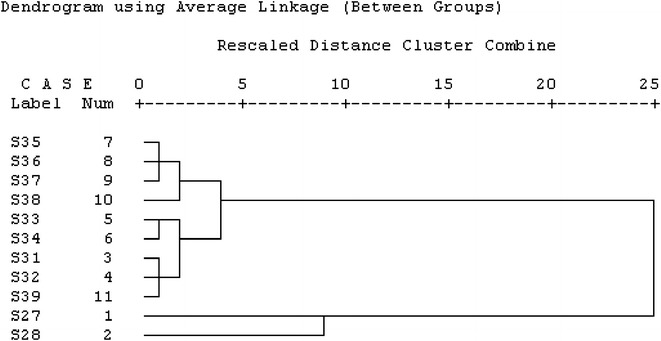
Dendrogram of clustering analysis for “Qin-Jiao” and its adulterant. (11 samples)

**Fig. 8:**
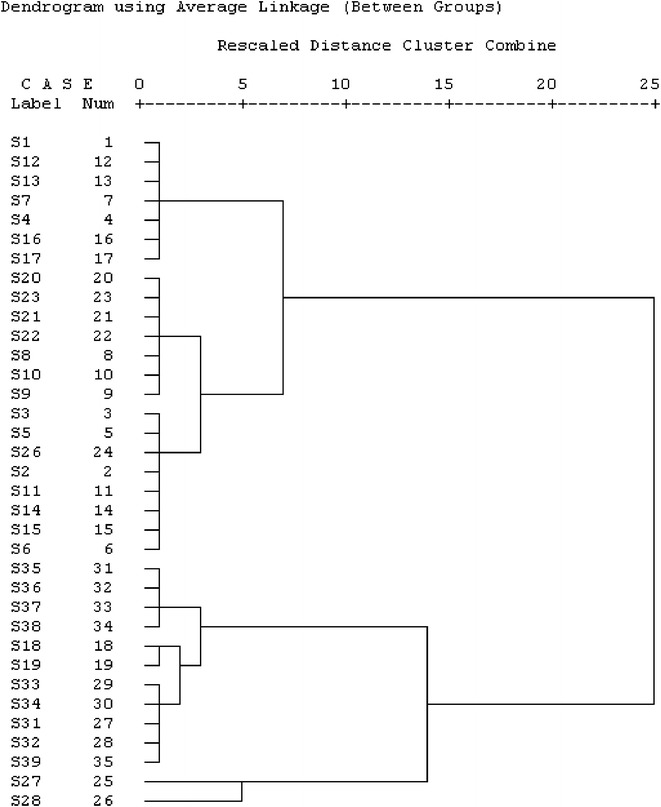
Dendrogram of clustering analysis for 35 samples

According to the fingerprint analysis, seven common characteristic peaks were found among 35 samples. The hierarchical clustering analysis of all the “Long-Dan” and “Qin-Jiao” samples with their adulterants were showed in Figs. 6, 7 and 8.

As shown in Fig. 8, two groups A with 22 samples and B with 13 samples were obtained from 35 batches of “Long-Dan”, “Qin-Jiao” and their adulterants samples. It was obvious that group B contained all the “Qin-Jiao” and its adulterant samples, as well as one of “Long-Dan” species, *G. triflora* (S18 and S19). In addition, most of the “Long-Dan” samples and all of its adulterants samples were into group A. It was observed in group B that all species, e.g. *G. crassicaulis* (S27–S28), *G. straminea* (S31–S34), *G. dahurica* (S35–S37) and *G. triflora* (S18–S19), were clearly separated from each other, except that *G. macrophylla* (S38) and *G. robusta* (S39) were mixed into *G. dahurica* and *G. straminea* respectively (Figs. 6 and 8). According to group A, only the samples of *G. purdomii* (S20–S23) were categorized into together, the left samples especially the samples of *G. rigescens* (S1–S16) distributed a little mass and the samples of *G. scabra* and *G. microdonta* were not discriminated with other species, so was the result of Fig. 7. The result indicated that more number of samples and data of characteristic peaks were needed to improve a more comprehensive and accurate categorization. Though the grouping of 35 samples of “Long-Dan”, “Qin-Jiao” and their adulterants in hierarchical clustering analysis was not all well in agreement with the species, it supported that *G. purdomii*, *G. microdonta*, and *G. robusta* could be used as the adulterants of “Long-Dan” and “Qin-Jiao”, respectively.

### Principal Component Analysis (PCA)

**Fig. 9:**
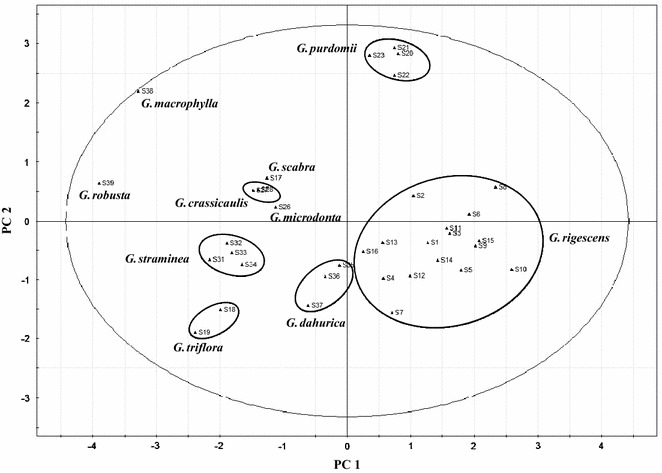
The PCA *score plots* for 35 samples using combination of PC 1 and PC 2

PCA is a kind of a clustering statistical method which reduces the dimensionality of multivariate data to express the original variables as a particular linear combination of the principal components (PCs) in the score plots. Moreover, the plotted data can enhance the visualization of similarities and differences in the data set, allowing for improved discrimination among samples [18, 19]. The relationship of “Long-Dan”, “Qin-Jiao”, and their adulterants from 10 *Gentiana* species was investigated on by PCA using the data of seven common peaks 1–7. As shown in Fig. 9. Ten *Gentiana* species could be clearly discriminated in the score plots constructed by combining PC 1 (41.5 %) and PC 2 (23.2 %). From the score plots, most of the “Qin-Jiao” and “Long-Dan” species were separated by PC1 whereas some samples from “Long-Dan” species, e.g. S17 (*G. scabra*) and S18-S19 (*G. triflora*) were in the area of “Qin-Jiao”. The result indicated that “Long-Dan” and “Qin-Jiao” could not be discriminated from each other by using these seven common peaks in the present study. And this might be the reason that “Long-Dan”, “Qin-Jiao” and their adulterants have been easily confused by the local people. From the phytochemical point of view, it is important to increase the characteristic components of “Long-Dan” and “Qin-Jiao”, in order to distinguish them reasonably.

## Experimental

### General

Loganic acid (**1**) and 2′-(*o,m*-dihydroxybenzyl)sweroside (**5**) were isolated by our laboratory and confirmed by NMR and MS spectroscopy for structures [20, 21] and HPLC for purity (>98 %). Swertiamarinin (**2**), gentiopicroside (**3**), and sweroside (**4**) were bought from the National Institute for the Control of Pharmaceutical and Biological (NICPBP). MeOH (chromatographic grade), acetonitrile (chromatographic grade) and phosphoric acid (reagent grade) were purchased from Merck (Darmstadt, Germany). Water was purified with a Milli-Qapparatus (Millipore, Bedford, MA). RC membrane filters, 0.45 μm, Φ 25 mm, were purchased from IVA (Meerbusch, Germany).

### Plant Material

The studied plant materials (Talbe 1 of ESM) included 26 “Long-Dan” samples from three officinal species of *G. rigescens* (S1–S16), *G. scabra* (S17), *G. triflora* (S18–S19), and four adulterants including *G. purdomii* (S20–S23), *G. erecto*-*sepala* (S24), *G. obconica* (S25) and *G. microdonta* (S26), and 13 “Qin-Jiao” samples from four officinal species, e.g. *G*. *crassicaulis* (S27–S30), *G. straminea* (S31–S34), *G. dahurica* (S35–S37) and *G. macrophylla* (S38), and one related adulterant, *G. robusta* (S39). Since *G. manshurica*, one of the “Long-Dan” officinal species is tending to extinguish and hard for collecting in the open field, it is lacking in the sample list.

The samples were collected in southwestern China (Yunnan and Sichuan provinces) for *G*. *rigescens*, *G*. *purdomii*, *G*. *crassicaulis*, and *G*. *microdonta*, in northeastern China (Jilin and Liaoning provinces) for *G. scabra* and *G. triflora*, in southwestern and northwestern China (Tibet, Qinghai and Gansu provinces) for *G. straminea*, *G. dahurica*, and *G. macrophylla*, and in Tibet for *G. robusta*, *G. erecto*-*sepala*, *and G. obconica*, respectively. All of the plant materials were collected from February to June of 2011. The botanical origins of all the collected samples were identified by Dr. Shu-Dong Zhang and Rong Li from Kunming Institute of Botany (KIB), Chinese Academy of Sciences (CAS), during the field collection. The specimens of all these materials were deposited at the State Key Laboratory of Phytochemistry and Plant Resources in West China, KIB, CAS. The voucher numbers were shown in Table 1 of ESM (SI1).

### HPLC and HPLC–MS Analysis

The powdered roots (0.25 g) of each sample were immersed in MeOH (10 mL) over eight hours and then extracted under ultrasonic condition for 30 min. The obtained residue was filtered through a syringe filter (0.45 μm), and an aliquot of each filtrate (10 μL) was injected into the HPLC instrument for analysis. HPLC analysis was performed on an Agilent series 1260 (Agilent Technologies) liquid chromatography, equipped with a vacuum degasser, a quaternary pump, an autosampler, and a diode array detector (DAD). An Agilent ZORBAX SB-C_18_ column (4.6 × 150 mm, 5 μm) was used. The following gradient system was used with water containing 0.2 % (v/v) H_3_PO_4_ (solvent A) and acetonitrile (solvent B): 0–25 min: linear 8–20 % of B; 25–26 min: linear 20–100 % of B. The flowing rate was 1 mL/min, and the detection wavelength was at 254 nm. Diode array detection was between 190 and 650 nm and the column temperature was set at 40 °C and the monitored wavelength was 254 nm.

HPLC–DAD–MS analysis was performed on a Agilent series 1100 (Agilent Technologies) liquid chromatography, equipped with a vacuum degasser, a quaternary pump, an autosampler, and a DAD and an ion-trap mass spectrometer with electrospray interface (ESI), operating in full scan MS mode from 150 to 1,500 amu. Samples were analyzed using both negative and positive ionization modes. ESI–MS parameters were as follows: potential of the ESI source, 4 kV; capillary temperature, 400 °C. An Agilent ZORBAX SB-C_18_ column (4.6 × 150 mm, 5 μm) was used. The mass traces of five were recorded, and identification of individual compounds was conducted by MS^n^ ragmentation and comparison with standards. The gradient system was the same system as described in the above HPLC conditions part. HPLC injection volume was 10 μL. The result was shown in Figs. 2 and 3 of ESM (SI5 and SI6).

### Calibration of Compounds **1**–**5**

Standard samples of compounds **1**–**5** were prepared into appropriate concentration, and the calibration curve for each compound was performed with six different added quantities in triplicate by plotting the peak area versus the quantities of the compounds. All five calibration curves exhibited good linear regressions, and the results are shown in Table 2 and Fig. 1 of ESM (SI2 and SI3).

### Method Evaluation

Selectivity was determined by comparing the chromatograms obtained from the *Gentiana* samples with those of the standard solutions. Precision was calculated in terms of intra-day (*n* = 6) with the standard solution of compounds **1**–**5** on the Agilent ZORBAX SB-C_18_ column and evaluated by calculating the relative standard deviation (RSD). In order to test the repeatability, solutions of sample 1 were prepared and it was injected 6 times (Table 3 of ESM, SI4). Other method evaluation was performed as described by our previous studies [22].

### Data Analysis

A professional and recommended software by the SFDA of China, named Similarity Evaluation System for Chromatographic Fingerprint of TCM (Version 2004 A) was used for similarity analysis of chromatographic profiles of “Long-Dan”, “Qin-Jiao” and their adulterants. By which, seven common peaks in the chromatograms were selected and the peak of gentiopicroside (**3**) was used as the reference. The relative retention time (RRT) and RPA of each common peak to the reference in the chromatograms were calculated. The hierarchical clustering analysis (HCA) of 35 samples was performed with between-group linkage method in SPSS (version 16.0, USA). In addition, principal component analysis (PCA) was also applied to clarify the relationship between these species by using SIMCA-P (version 11.0 Umetrics, Umea, Sweden).

## Conclusions

A validated HPLC–UV method for simultaneously quantifying of five iridoid glycosides, e.g. loganic acid (**1**), swertiamarinin (**2**), gentiopicroside (**3**), sweroside (**4**) and 2′-(*o*,*m*-dihydroxybenzyl)sweroside (**5**), in “Long-Dan”, “Qin-Jiao” and their adulterants was established in the present study. It was found that the chemical constituents of “Long-Dan”, “Qin-Jiao” and their adulterants were differed from each other, even among the samples from the same species, due to different geographical positions and climatic conditions, which may cause the qualitative differences between the plants from various areas.

In the Chinese Pharmacopoeia, it recorded that the content of gentiopicroside (**3**) should be no less than 2 % in “Long-Dan” with an exception for *G. rigescens* (no less than 1 %), and the total contents of gentiopicroside (**3**) and loganic acid (**1**) must be no less than 2.5 % in “Qin-Jiao”. Our present study showed that except for loganic acid (**1**) and gentiopicroside (**3**), other two iridoid glycosides, swertiamarinin (**2**) and sweroside (**4**) were also common constituents in “Long-Dan” and “Qin-Jiao”, while 2′-(*o*,*m*-dihydroxybenzyl)sweroside (**5**) was only detected in one “Long-Dan” species, *G. rigescens*. Swertiamarinin (**2**), sweroside (**4**), and 2′-(*o,m*-dihydroxybenzyl)sweroside (**5**) were reported to have potential COX1/2 inhibition in zebrafish model [12]. It suggested that their contents should be also used for the quality control of “Long-Dan” and “Qing-Jiao”, which maybe more accurate if multivariate quantitative detection of these bioactive ingredients as control was adapted. Moreover, from the phytochemical point of view, our study supported that the four different *Gentiana* species have been recorded as one of the “Long-Dan” or “Qin-Jiao” origins in the Chinese Pharmacopoeia. Although the contents of compounds **1**–**5** were various in different species, it should explain the geo-herbalism opinion in phytochemical terms. 

## Electronic Supplementary Material

The sample list, the contents (%), calibration curves, ^1^H NMR and MS spectra of compounds **1**–**5**, the intraday precision of sample 1 (S1), the HPLC–MS spectra of compounds **1**–**5** in sample 1 (S1) are provided as links available below as supporting information.

## Electronic supplementary material

Below is the link to the electronic supplementary material.

Supplementary material 1 (DOCX 3366 kb)
