# Supplementary material for: Comparative Study on “Long-Dan”, “Qin-Jiao” and Their Adulterants by HPLC Analysis
Source: Nat Prod Bioprospect. 2014 Oct 4;4(5):297–308. doi: 10.1007/s13659-014-0039-x (PMC4199948; doi:10.1007/s13659-014-0039-x)

**Supplementary data contents page**

***Title:* *Comparative study on "Long-Dan", "Qin-Jiao" and their adulterants by HPLC analysis***

***Authors:*** Fang-Fang Liu ^a,c,d^, Yan-Ming Wang ^a,b,d^, Hong-Tao Zhu ^a^, Dong Wang ^a^, Chong-Ren Yang ^a^ Min Xu *^a^ and Ying-Jun Zhang *^a^

**Address:** *^a^ State Key Laboratory of Phytochemistry and Plant Resources in West China, Kunming Institute of Botany, Chinese Academy of Sciences, Kunming 650204, P. R. China*

*^b^ University of Chinese Academy of Sciences, Beijing 100049, P. R. China*

*^c^ Yunnan University of Traditional Chinese Medicine, Kunming 650500, P. R. China*

*^d^ The authors contributed equally to this paper*

SI1 Table 1 Sample list and contents (%) of loganic acid, swertiamarinin, gentiopicroside, sweroside and 2'-(*o*,*m*-dihydroxybenzyl)sweroside

SI2 Table 2 Calibration curves for standard compounds

SI3 Figure 1 Calibration curves for standard compounds

SI4 Table 3 Intraday precision of sample 1 (S1)

SI5 Figure 2 The HPLC-MS spectra of five major compounds in sample 1 (S1)

SI6 Figure 3 ^1^H-NMR and MS spectra of loganic acid, swertiamarinin, gentiopicroside, sweroside and 2'-(*o*,*m*-dihydroxybenzyl)sweroside

SI1 Table 1. Sample list and contents (%) of five major compounds

| No. | Species | Origins | Voucher number | Loganic acid | Swertiamarinin | Gentiopicroside | Sweroside | 2'-(*o*,*m*-dihydroxybenzyl) sweroside |
| --- | --- | --- | --- | --- | --- | --- | --- | --- |
| S1 | *G. rigescens* | Dali,Yunnan | KUN_552246 | 0.429±0.009 | 0.067±0.003 | 2.127±0.042 | 0.161±0.004 | 0.143±0.002 |
| S2 | *G. rigescens* | Dali,Yunnan | KUN_552265 | 0.585±0.005 | 0.076±0.004 | 2.311±0.035 | 0.148±0.003 | 0.094±0.001 |
| S3 | *G. rigescens* | Dali,Yunnan | KUN_552266 | 0.341±0.004 | 0.026±0.006 | 2.273±0.041 | 0.138±0.002 | 0.215±0.007 |
| S4 | *G. rigescens* | Dali,Yunnan | KUN_552267 | 0.453±0.002 | 0.070±0.002 | 2.654±0.007 | 0.125±0.002 | 0.017±0.001 |
| S5 | *G. rigescens* | Kunming, Yunnan | KUN_552275 | 0.111±0.002 | 0.036±0.004 | 2.367±0.067 | 0.087±0.001 | 0.285±0.005 |
| S6 | *G. rigescens* | Kunming, Yunnan | KUN_552279 | 0.344±0.008 | 0.023±0.003 | 1.752±0.017 | 0.096±0.003 | 0.224±0.004 |
| S7 | *G. rigescens* | Kunming, Yunnan | KUN_552282 | 0.311±0.009 | 0.032±0.005 | 3.499±0.187 | 0.125±0.002 | 0.276±0.005 |
| S8 | *G. rigescens* | Lijiang, Yunnan | IBSC_0490155 | 0.187±0.005 | 0.018±0.002 | 1.157±0.041 | 0.141±0.001 | 0.152±0.003 |
| S9 | *G. rigescens* | Lijiang, Yunnan | IBSC_0490156 | 0.408±0.007 | 0.016±0.001 | 1.035±0.015 | 0.132±0.004 | 0.240±0.005 |
| S10 | *G. rigescens* | Wenshan, Yunnan | KUN_552257 | 0.043±0.006 | 0.016±0.002 | 1.066±0.006 | 0.051±0.002 | 0.138±0.003 |
| S11 | *G. rigescens* | Wenshan, Yunnan | KUN_552309 | 0.436±0.011 | 0.090±0.005 | 2.082±0.036 | 0.349±0.008 | 0.099±0.002 |
| S12 | *G. rigescens* | Wenshan, Yunnan | KUN_552314 | 0.239±0.006 | 0.042±0.003 | 2.778±0.102 | 0.106±0.005 | 0.149±0.003 |
| S13 | *G. rigescens* | Wenshan, Yunnan | KUN_552315 | 0.424±0.004 | 0.054±0.001 | 3.058±0.056 | 0.137±0.005 | 0.130±0.003 |
| S14 | *G. rigescens* | Wenshan, Yunnan | KUN_552340 | 0.263±0.006 | 0.051±0.004 | 1.945±0.062 | 0.131±0.006 | 0.183±0.004 |
| S15 | *G. rigescens* | Chuxiong, Yunnan | WUK_244813 | 0.373±0.003 | 0.008±0.001 | 1.533±0.059 | 0.086±0.003 | 0.144±0.006 |
| S16 | *G. rigescens* | Chuxiong, Yunnan | IBSC_0490156 | 0.510±0.005 | 0.059±0.002 | 2.389±0.142 | 0.105±0.002 | 0.064±0.002 |
| S17 | *G. scabra* | Changchun, Jilin | IBSC_0490287 | 0.710±0.008 | 0.290±0.001 | 2.820±0.038 | 0.268±0.007 | - |
| S18 | *G. triflora* | Qingyuan, Liaoning | WUK_187164 | 0.705±0.007 | 0.193±0.005 | 3.514±0.071 | 0.083±0.003 | - |
| S19 | *G. triflora* | Qingyuan, Liaoning | WUK_187548 | 0.420±0.009 | 0.230±0.003 | 4.773±0.059 | 0.084±0.003 | - |
| S20 | *G. purdomii* | Ganzi,Sichuan | CDBI_0115864 | 0.821±0.005 | 0.104±0.002 | 1.619±0.097 | 0.190±0.002 | - |
| S21 | *G. purdomii* | Ganzi,Sichuan | CDBI_0115865 | 0.228±0.005 | 0.062±0.004 | 1.375±0.061 | 0.132±0.004 | 0.020±0.002 |
| S22 | *G. purdomii* | Daofu,Sichuan | CDBI_0115866 | 0.179±0.004 | 0.088±0.005 | 2.007±0.080 | 0.434±0.009 | 0.182±0.007 |
| S23 | *G. purdomii* | Daofu,Sichuan | CDBI_0115868 | 0.156±0.004 | 0.137±0.002 | 3.635±0.081 | 0.508±0.012 | 0.094±0.002 |
| S24 | *G. erecto-sepala* | Linzhi, Tibet | KUN_551171 | 0.126±0.003 | 0.073±0.003 | 0.707±0.009 | 0.160±0.003 | 0.061±0.001 |
| S25 | *G. obconica* | Linzhi, Tibet | PE_00088467 | 0.091±0.002 | 0.023±0.001 | 0.599±0.004 | 0.264±0.005 | - |
| S26 | *G. microdonta* | Diqing, Yunnan | KUN_551661 | 0.161±0.003 | 0.144±0.004 | 2.530±0.017 | 0.258±0.005 | 0.243±0.005 |
| S27 | *G. crassicaulis* | Diqing, Yunnan | KUN_550928 | 1.320±0.006 | 0.269±0.001 | 6.301±0.096 | 0.078±0.002 | - |
| S28 | *G. crassicaulis* | Wenshan, Yunnan | PE_00066285 | 0.213±0.004 | 0.297±0.003 | 5.411±0.080 | 0.157±0.003 | - |
| S29 | *G. crassicaulis* | Ganzi, Sichuan | PE_00066383 | 0.062±0.002 | 0.065±0.007 | 1.952±0.059 | 0.151±0.002 | - |
| S30 | *G. crassicaulis* | Ganzi, Sichuan | KUN_550921 | 0.043±0.001 | 0.052±0.004 | 0.611±0.006 | 0.042±0.004 | - |
| S31 | *G. straminea* | Changdu, Tibet | KUN_552663 | 0.865±0.006 | 0.307±0.002 | 4.198±0.057 | 0.595±0.008 | - |
| S32 | *G. straminea* | Changdu, Tibet | KUN_552672 | 0.715±0.004 | 0.263±0.006 | 4.014±0.111 | 0.211±0.006 | - |
| S33 | *G. straminea* | Changdu, Tibet | KUN_552673 | 0.692±0.003 | 0.249±0.005 | 3.983±0.101 | 0.761±0.013 | - |
| S34 | *G. straminea* | Xining, Qinghai | KUN_552659 | 0.270±0.005 | 0.101±0.003 | 2.285±0.033 | 0.141±0.004 | - |
| S35 | *G. dahurica* | Xining, Qinghai | HNWP_133660 | 0.272±0.004 | 0.188±0.002 | 3.929±0.132 | 0.445±0.010 | - |
| S36 | *G. dahurica* | Xining, Qinghai | HNWP_158816 | 0.223±0.003 | 0.122±0.002 | 2.477±0.101 | 0.265±0.003 | - |
| S37 | *G. dahurica* | Xihe, Gansu | KUN_551006 | 1.243±0.007 | 0.266±0.003 | 4.690±0.108 | 0.118±0.002 | - |
| S38 | *G. macrophylla* | Xihe, Gansu | PE_00074150 | 1.434±0.007 | 0.182±0.001 | 2.324±0.053 | 0.639±0.006 | - |
| S39 | *G. robusta* | Jiangda, Tibet | HNWP_66834 | 0.842±0.010 | 0.257±0.004 | 3.589±0.061 | 0.401±0.009 | - |

“–”, means too low to be determined.

SI2 Table 2. Calibration curves for standard compounds

| Compounds | Calibration | R^2^ | Linear range (mg/L) |
| --- | --- | --- | --- |
| Loganic acid | y = 642897x + 16.878, | 1.0000 | 82.0-4100 |
| Swertiamarinin | y = 878070x + 17.062 | 0.9993 | 18.0-1800 |
| Gentiopicroside | y = 991234x + 90.187 | 1.0000 | 21.3-1704 |
| Sweroside | y = 568170x + 1.6768 | 0.9999 | 24.0-1440 |
| 2'-(*o*,*m*-dihydroxybenzyl)sweroside | y = 2×10^6^x + 16.486 | 1.0000 | 31.0-3100 |

SI3 Figure 1. Calibration curves for standard compounds


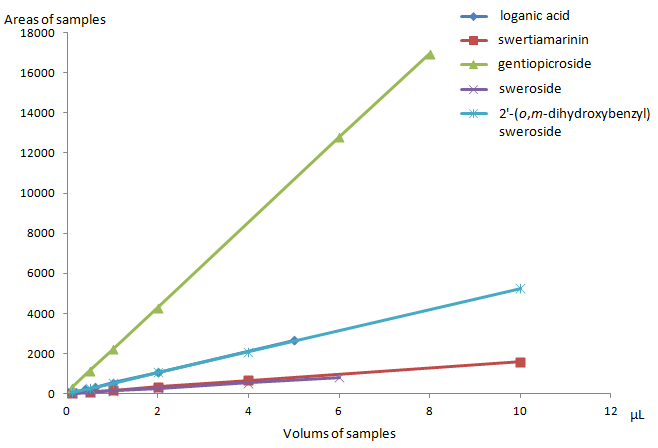


SI4 Table 3. Intraday precision (%) of sample 1 (S1)

| Compounds | Loganic acid | Swertiamarinin | Gentiopicroside | Sweroside | 2'-(*o*,*m*-dihydroxybenzyl) sweroside |
| --- | --- | --- | --- | --- | --- |
| Stability RSD | 1.35 | 3.21 | 1.33 | 2.22 | 3.33 |
| Repeatability RSD | 0.74 | 0.80 | 0.25 | 0.93 | 0.86 |

SI5 Figure 2 The HPLC-MS spectra of five major compounds in sample 1 (S1)

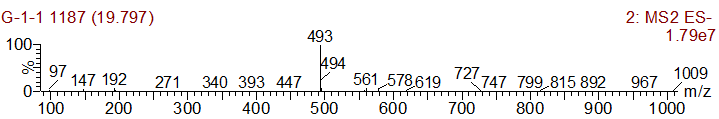

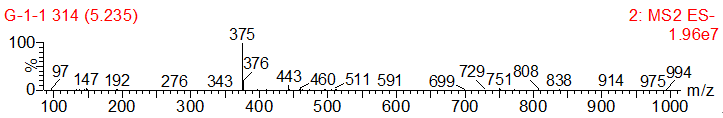


SI6 Figure 3 ^1^H-NMR and MS spectra of five major compounds

Loganic acid

**
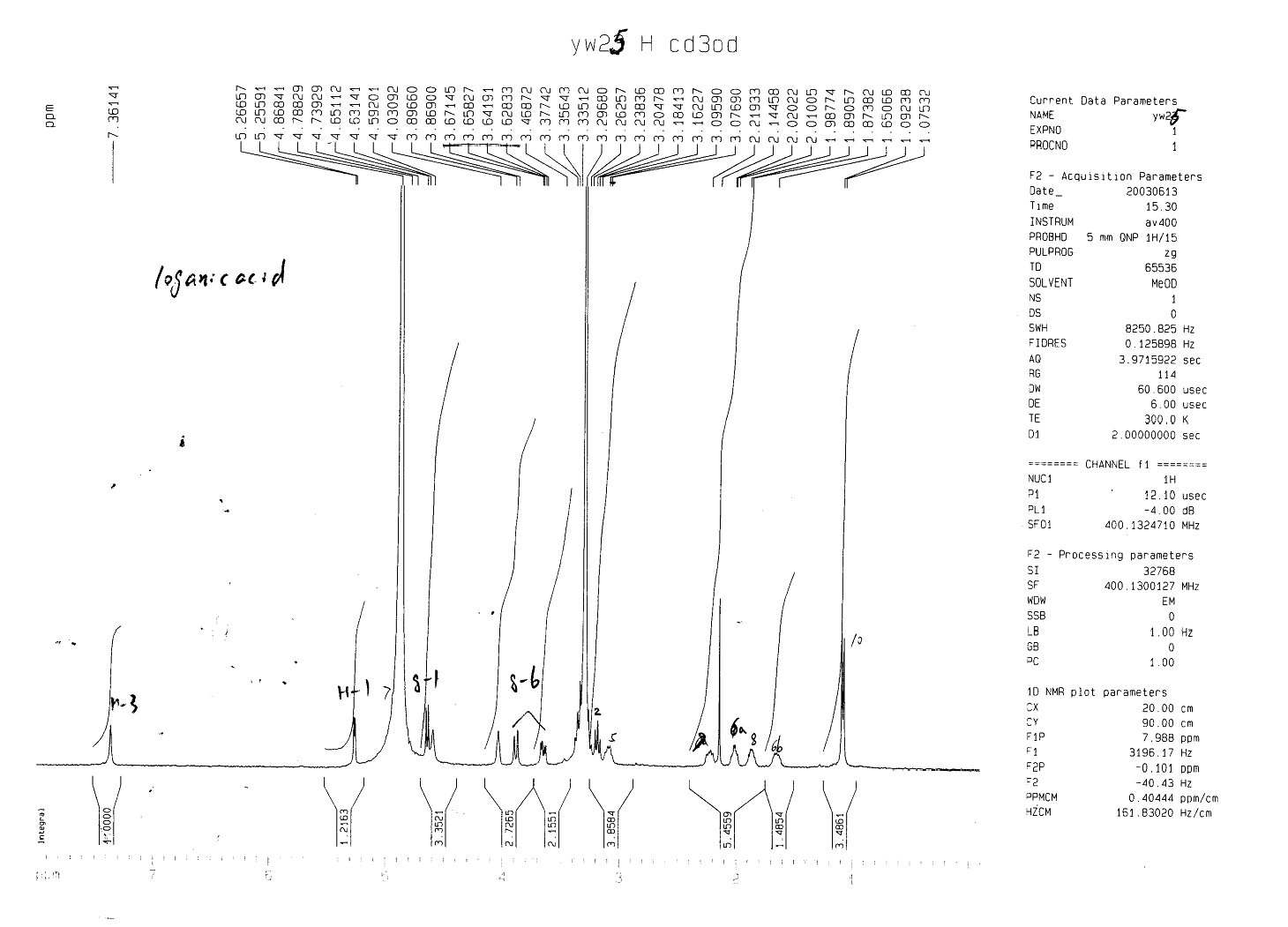
**

**
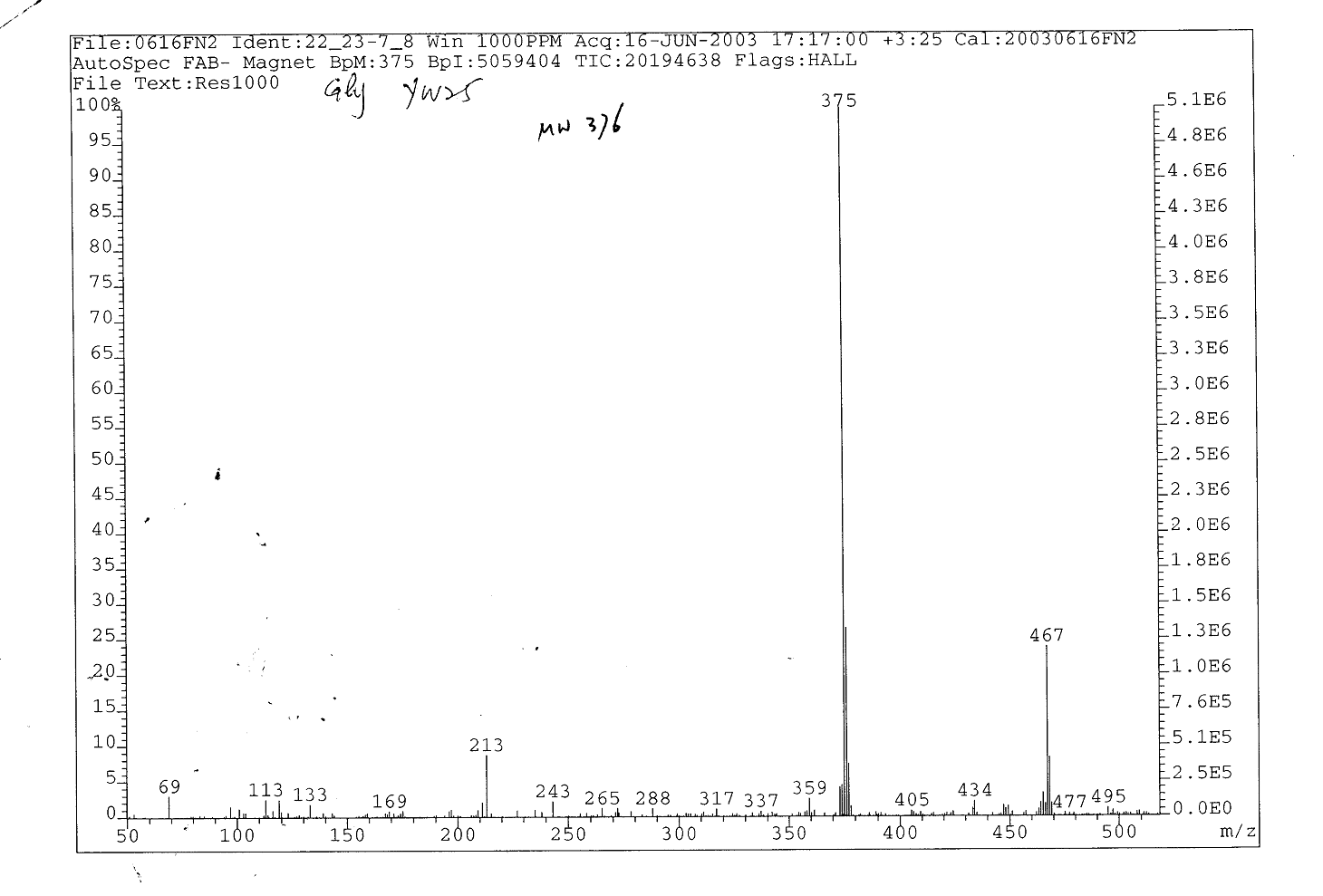
**

Swertiamarinin

**
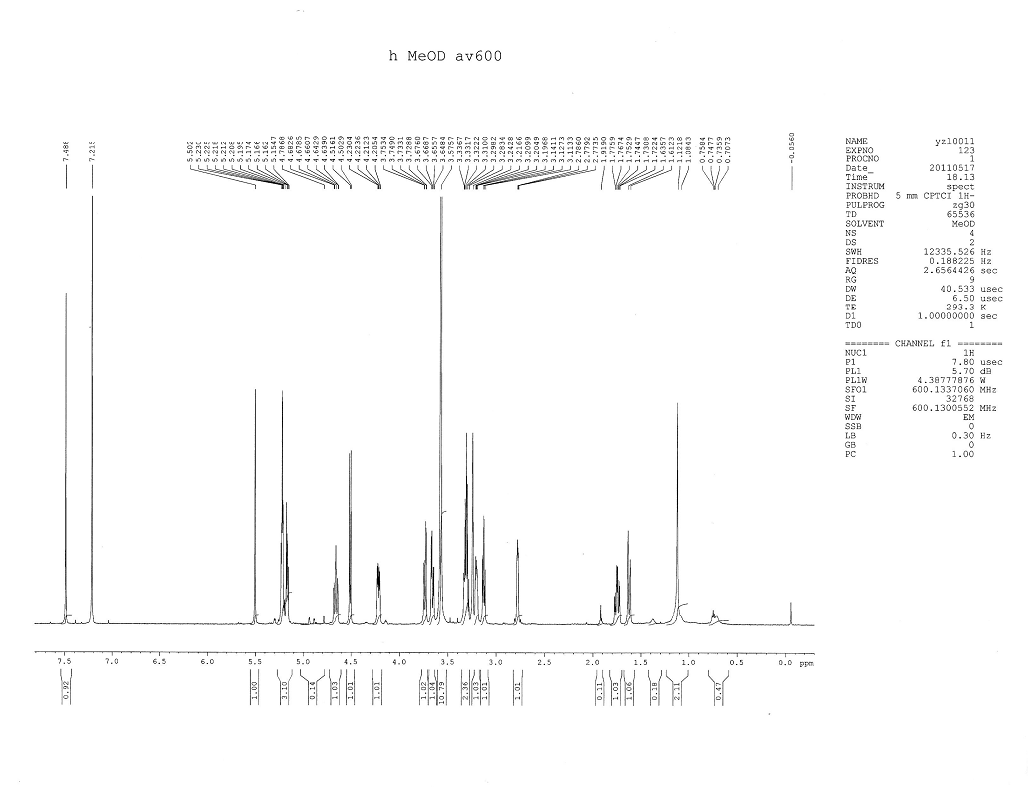
**

**
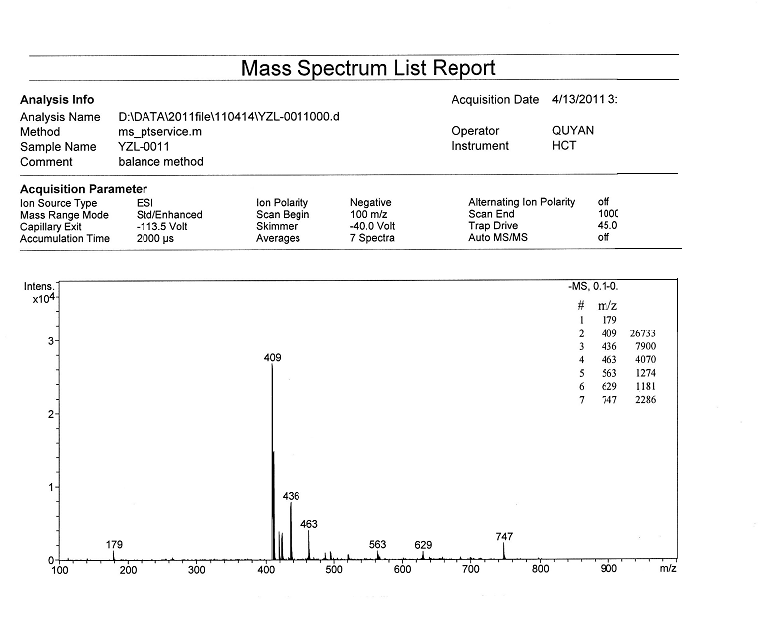
**

Gentiopicroside**
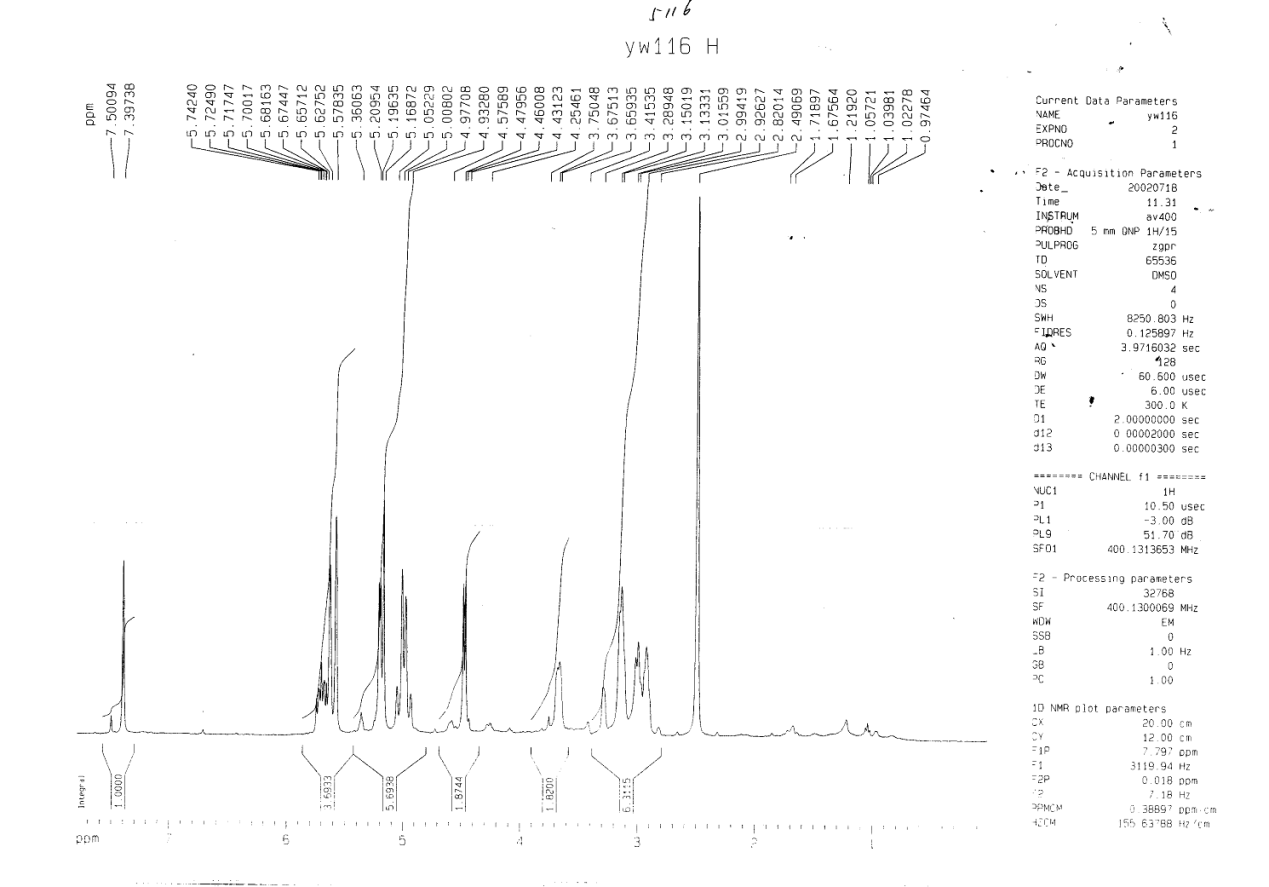
**

**
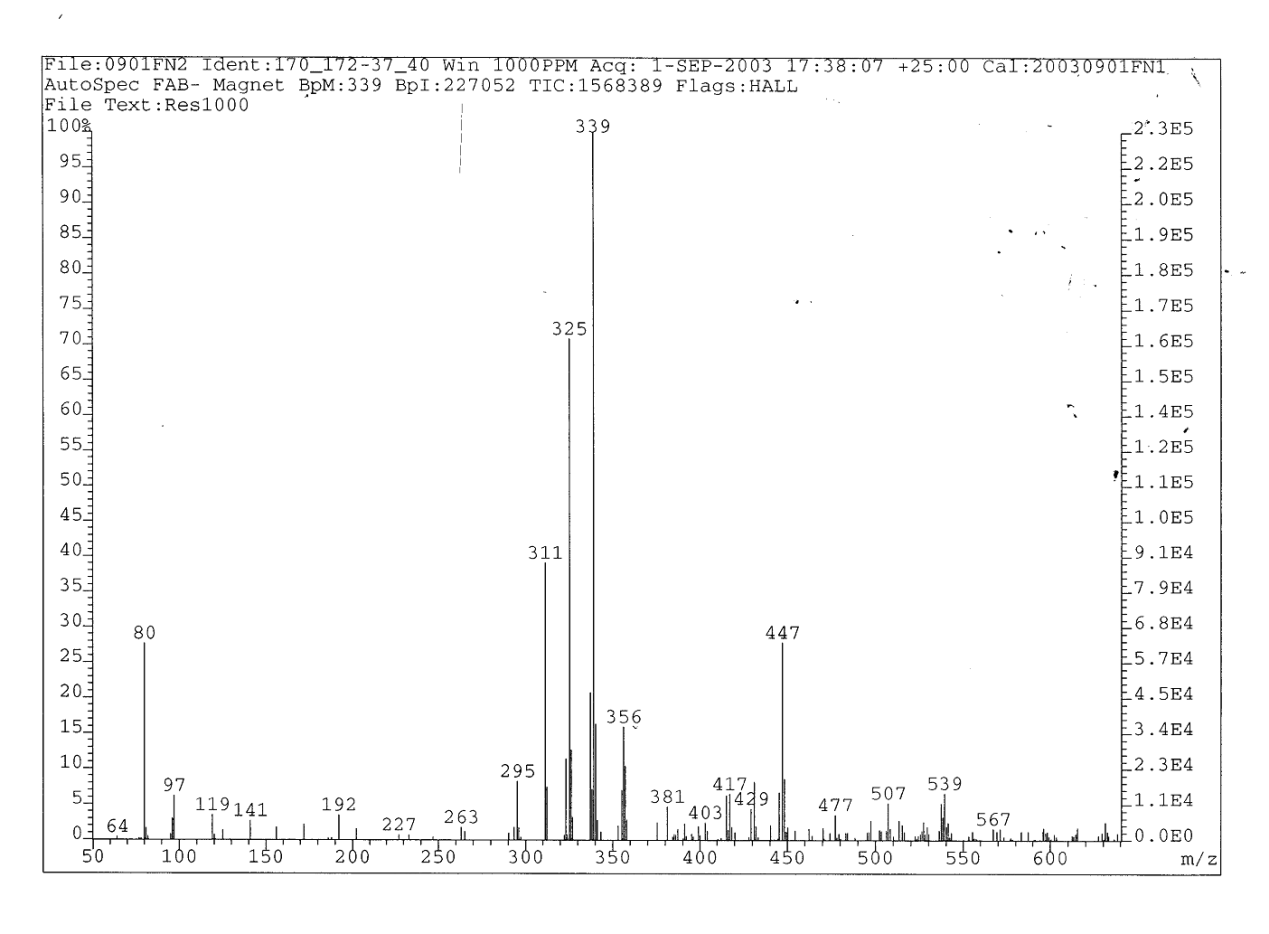
**

Sweroside

**
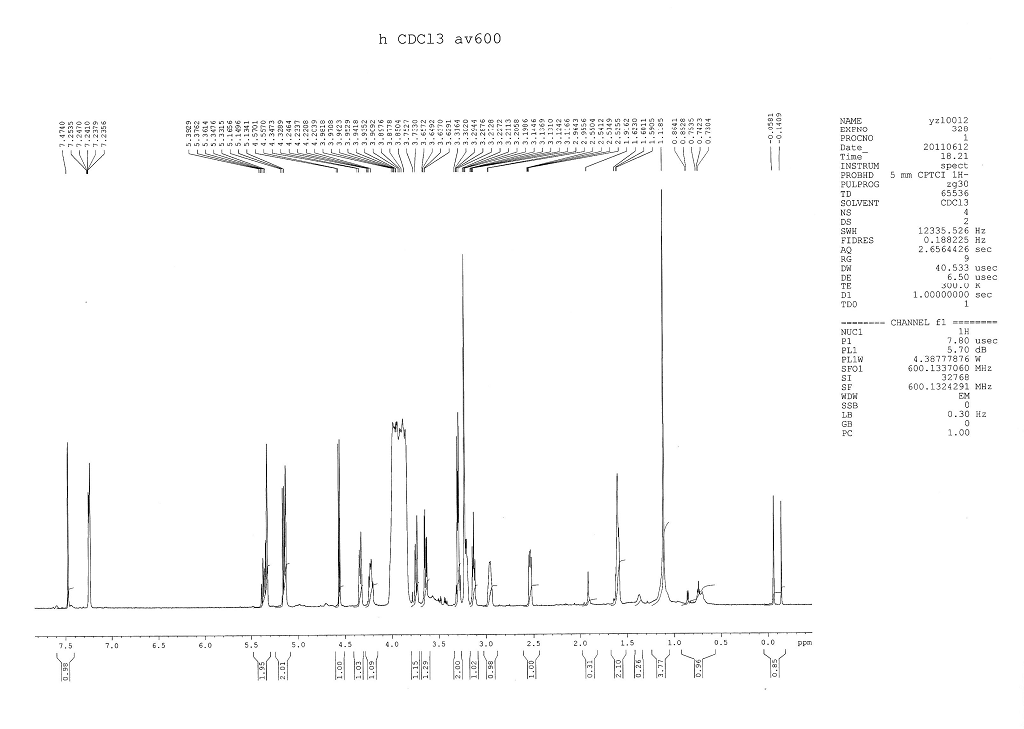
**

**
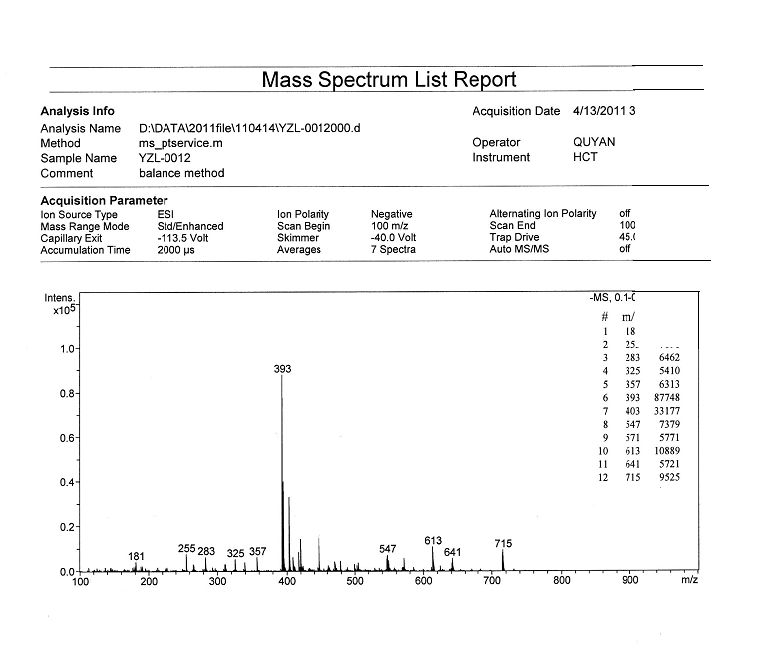
**

2'-(*o*,*m*-Dihydroxybenzyl)sweroside


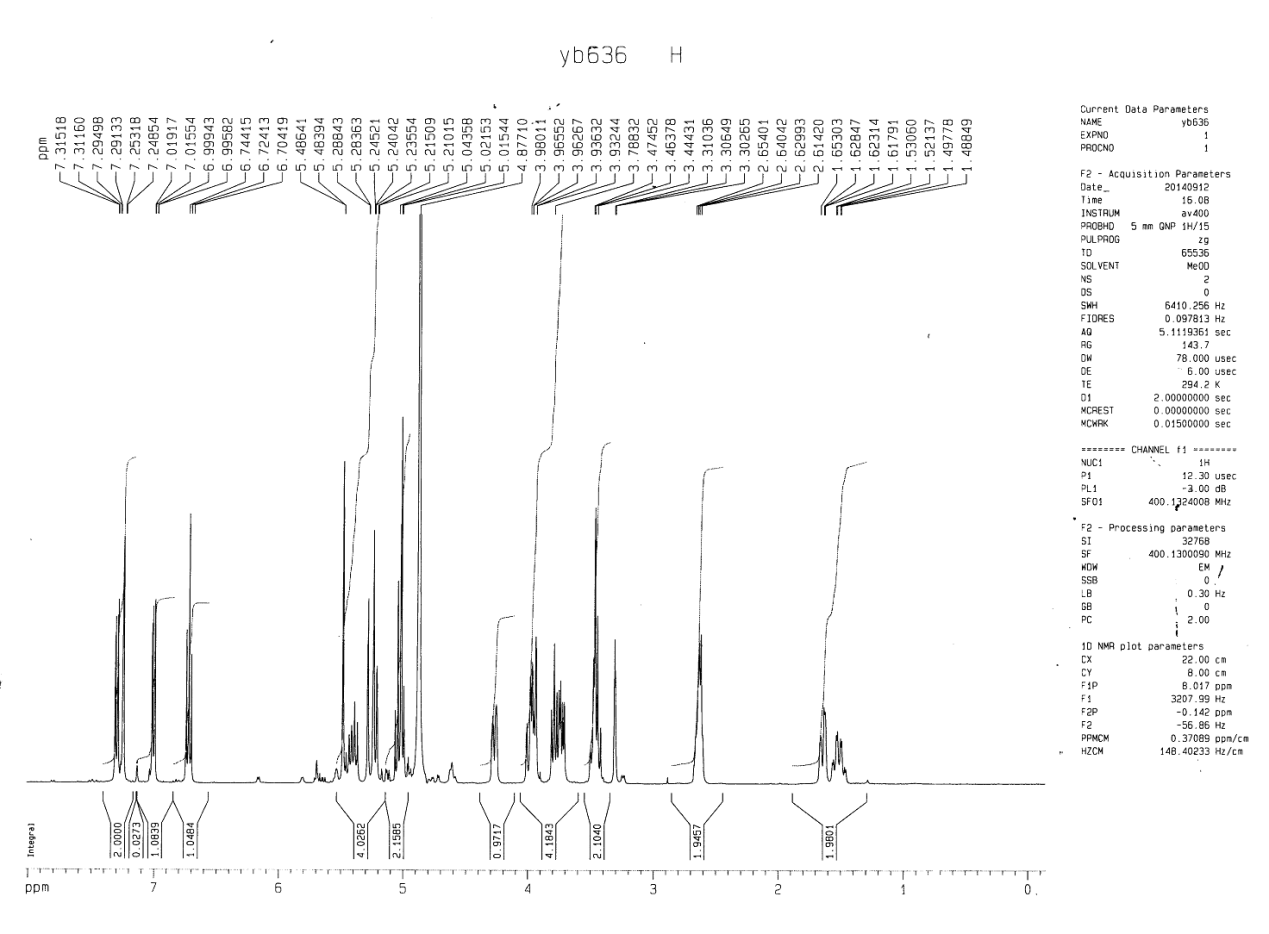


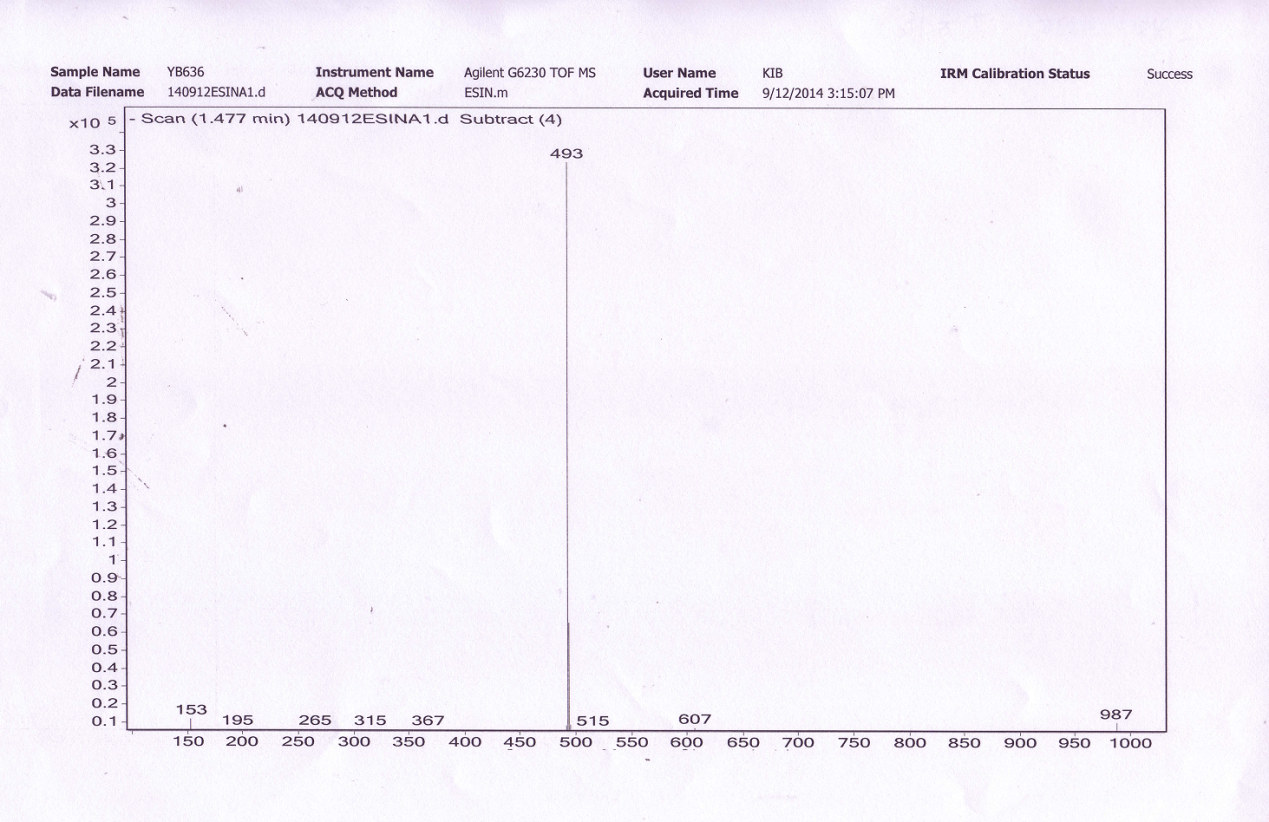

Supplement: Supplementary file 1 — Supplementary material 1 (DOCX 3366 kb) [file 13659_2014_39_MOESM1_ESM.docx]
